# Thermodynamics in Gliomas: Interactions between the Canonical WNT/Beta-Catenin Pathway and PPAR Gamma

**DOI:** 10.3389/fphys.2017.00352

**Published:** 2017-05-30

**Authors:** Alexandre Vallée, Yves Lecarpentier, Rémy Guillevin, Jean-Noël Vallée

**Affiliations:** ^1^Experimental and Clinical Neurosciences Laboratory, Institut National de la Santé et de la Recherche Médicale U1084, University of PoitiersPoitiers, France; ^2^Laboratoire de Mathématiques et Applications, UMR Centre National de la Recherche Scientifique 7348, Université de PoitiersPoitiers, France; ^3^Centre de Recherche Clinique, Hôpital de MeauxMeaux, France; ^4^DACTIM, Laboratoire de Mathématiques et Applications, Université de Poitiers et CHU de Poitiers, UMR Centre National de la Recherche Scientifique 7348, SP2MIFuturoscope, France; ^5^CHU Amiens Picardie, Université Picardie Jules VerneAmiens, France

**Keywords:** WNT/beta-catenin pathway, PPAR gamma, gliomas, circadian rhythms, aerobic glycolysis, Warburg effect, PI3K-Akt pathway, lactate

## Abstract

Gliomas cells are the site of numerous metabolic and thermodynamics abnormalities with an increasing entropy rate which is characteristic of irreversible processes driven by changes in Gibbs energy, heat production, intracellular acidity, membrane potential gradient, and ionic conductance. We focus our review on the opposing interactions observed in glioma between the canonical WNT/beta-catenin pathway and PPAR gamma and their metabolic and thermodynamic implications. In gliomas, WNT/beta-catenin pathway is upregulated while PPAR gamma is downregulated. Upregulation of WNT/beta-catenin signaling induces changes in key metabolic enzyme that modify their thermodynamics behavior. This leads to activation pyruvate dehydrogenase kinase 1(PDK-1) and monocarboxylate lactate transporter 1 (MCT-1). Consequently, phosphorylation of PDK-1 inhibits pyruvate dehydrogenase complex (PDH). Thus, a large part of pyruvate cannot be converted into acetyl-CoA in mitochondria and in TCA (tricarboxylic acid) cycle. This leads to aerobic glycolysis despite the availability of oxygen, named Warburg effect. Cytoplasmic pyruvate is, in major part, converted into lactate. The WNT/beta-catenin pathway induces also the transcription of genes involved in cell proliferation, cell invasiveness, nucleotide synthesis, tumor growth, and angiogenesis, such as c-Myc, cyclin D1, PDK. In addition, in gliomas cells, PPAR gamma is downregulated, leading to a decrease in insulin sensitivity and an increase in neuroinflammation. Moreover, PPAR gamma contributes to regulate some key circadian genes. Abnormalities in the regulation of circadian rhythms and dysregulation in circadian clock genes are observed in gliomas. Circadian rhythms are dissipative structures, which play a key role in far-from-equilibrium thermodynamics through their interactions with WNT/beta-catenin pathway and PPAR gamma. In gliomas, metabolism, thermodynamics, and circadian rhythms are tightly interrelated.

## Introduction

Gliomas are the most frequent primary brain tumors. Around 30 percent of all brain and central nervous system tumors and 80 percent of all malignant brain tumors are gliomas. They are characterized by their infiltrating nature, especially into the surrounding normal brain tissue (Goodenberger and Jenkins, 2012). Glial cells have the potency to divide and multiply, and failure in control of this potency may result in the formation of a glioma. They contain multipotent tumor stem cells, which have the potential to be transformed into variants of normal neural progenitor cells (Galli et al., 2004; Singh et al., 2004). Gliomas are named on the cell type with which they share histological characteristics. They are named astrocytomas (multiform glioblastoma), oligodendrogliomas, ependymomas, and mixed gliomas (oligoastrocytomas) based on their resemblance with astrocytes, oligodendrocytes, ependymal cells, and mixed glial cells, respectively. Gliomas are classified into grade I to IV based on the criteria set by World Health Organization, with a higher-grade corresponding to more aggressive tumors. Grade I and grade II gliomas are slow-growing less aggressive tumors, whereas grade III and grade IV gliomas are malignant tumors characterized by high proliferation rate (grade III) and angiogenic activity (grade IV, Glioblastoma). Malignant gliomas are the most frequent malignant primary brain tumors (Louis, 2006; Mamelak and Jacoby, 2007; Ricard et al., 2012) and the most lethal human cancers (Patil et al., 2013). Glioblastoma patients present a median overall survival of 15 months, despite recent advances in diagnosis and therapy (Rouach et al., 2008). Prognosis of glioblastoma is still dismal. Therefore, it is essential to investigate the mechanisms underlying the development and progression of gliomas and to explore more effective therapeutic strategies.

Glioma cells are the sites of numerous metabolic and thermodynamic abnormalities. They are exergonic processes in which heat flows from the tumor to their surroundings (Gillies and Gatenby, 2015). The entropy rate increases in glioma cells and is characteristic of irreversible processes driven by changes in Gibbs energy, heat production, intracellular acidity, membrane potential gradient, and ionic conductance (Prigogine et al., 1974; Prigogine, 1986; Kondepudi and Prigogine, 1999; Hanselmann and Welter, 2016). Several cellular mechanisms can induce and develop carcinogenic processes.

WNT/beta-catenin signaling is a crucial factor in the development of many cancers (Leushacke and Barker, 2012; Ramachandran et al., 2012; Schepeler et al., 2012). Aberrant WNT/beta-catenin signaling has a key role in the development of glioblastoma (Palos et al., 1999), including cell proliferation (Pulvirenti et al., 2011), cell apoptosis inhibition (Satoh and Kuroda, 2000), and cell invasion (Roth et al., 2000).

In numerous tissues, canonical WNT/beta-catenin pathway activation induces inactivation of peroxisome proliferator-activated receptor gamma (PPAR gamma), while the activation of PPAR gamma induces inhibition of canonical WNT/beta-catenin pathway (Elbrecht et al., 1996; Fajas et al., 1997; Wang et al., 2008; Lecarpentier et al., 2010; Siersbæk et al., 2012; Zhang et al., 2012). WNT/beta-catenin is upregulated in glioma tissues in comparison with normal brain tissues, while PPAR gamma is downregulated (Wan et al., 2011). In glioma cells, upregulation of the WNT/beta-catenin signaling induces changes in key metabolic enzymes that modify their thermodynamics behavior. This leads to activation of pyruvate dehydrogenase kinase-1 (PDK-1) and monocarboxylate lactate transporter-1 (MCT-1) (Bienz and Clevers, 2000; Pate et al., 2014). Consequently, phosphorylation of PDK-1 inhibits the pyruvate dehydrogenase complex (PDH). Thus, a large part of pyruvate cannot be converted into acetyl-coenzyme A (acetyl-CoA) in mitochondria and acetyl-CoA cannot enter the tricarboxylic acid (TCA) cycle. This leads to aerobic glycolysis despite the availability of oxygen. Cytoplasmic pyruvate is, in major part, converted into lactate. This phenomenon is referred to as the Warburg effect (Warburg, 1956). Glioblastomas present a metabolic remodeling (Tsacopoulos and Magistretti, 1996) with an increase of both aerobic glycolysis and lactate production (Moon et al., 2011). Increased lactate production is associated with increased aggressiveness, angiogenesis, and poor prognosis (Gruetter, 2003; Keenan and Chi, 2015). The WNT/beta-catenin pathway induces the transcription of genes involved in cell proliferation (c-Myc, cyclin D1, PDK). This ultimately promotes the nucleotide, protein and lipid synthesis necessary for cell growth and multiplication.

PPAR gamma is downregulated in glioma cells while PPAR gamma contributes to regulate some key circadian genes. Circadian rhythms (CRs) are dissipative structures, which play a key role in far-from-equilibrium thermodynamics. In gliomas, abnormalities in the regulation of CRs are observed (Fujioka et al., 2006; Yang et al., 2011; Li et al., 2013). PPAR dysfunction influences statistical mechanics by modifying thermodynamic force, thermodynamic flow, and rate of entropy production (Lecarpentier et al., 2008).

From a thermodynamic viewpoint and among numerous cellular processes involved in gliomas, the opposite profile of the canonical WNT/beta-catenin pathway and PPAR gamma in gliomas play a key role in both aerobic glycolysis (Warburg effect) and disruption of circadian rhythms. The thermodynamic dysregulation induced by these two processes is consubstantial with metabolic abnormalities found in glioma.

We focus this review on the opposing interactions observed in glioma between the canonical WNT/beta-catenin pathway and PPAR gamma and their metabolic and thermodynamic implications.

## Canonical WNT/beta-catenin pathway

The canonical WNT/beta-catenin pathway plays an important role in metabolism, embryonic development, cell fate, and epithelial-mesenchymal transition (EMT). The canonical WNT activity is reflected by elevated levels of beta-catenin in the nucleus and/or cytoplasm, which can be detected by means of immunohistochemical staining and Western blotting. Its dysfunction is involved in numerous diseases, particularly in cancers (Moon et al., 2002, 2004; Nusse, 2005; Clevers, 2006), such as gliomas (Utsuki et al., 2002; Sareddy et al., 2009; Yang et al., 2010; Liu C. et al., 2011; Liu X. et al., 2011; Rossi et al., 2011; Kahlert et al., 2012; Schule et al., 2012; Shi et al., 2012; Yang C. et al., 2012; Denysenko et al., 2016; Lee et al., 2016). WNT pathway is a transcriptional program driven by beta-catenin/T-cell/lymphoid enhancer (TCF/LEF). The destruction complex consists of Axin, tumor suppressor adenomatous polyposis coli (APC), and glycogen synthase kinase-3 (GSK-3beta). It exerts a tightly control on the beta-catenin pathway. In the absence of WNT ligands (“off state”), the destruction complex phosphorylates beta-catenin, which is then degraded in the proteasome. In the presence of WNT ligands (“on state”), the WNT receptor interacts with Frizzled (Fzl) and LDL receptor-related protein 5/6 (LRP 5/6). WNT receptor is associated with Dishevelled (Dsh). This triggers the disruption of the destruction complex and prevents degradation of beta-catenin in the proteasome. Beta-catenin then translocates to the nucleus and interacts with TCF/LEF. This leads to the stimulation of beta-catenin target genes (PDK, MCT-1, c-Myc, cyclin D1, Cox 2, Axin2…) (He et al., 1998; Shtutman et al., 1999; Angers and Moon, 2009; Pate et al., 2014). In glioma cells, overexpression of c-Myc, a WNT target gene, promotes the Warburg effect via activation of downstream genes, such as glucose transporter (Glut), hexokinase (HK), pyruvate dehydrogenase kinase 1 (PDK1), and lactate dehydrogenase A (LDH-A) (Wang et al., 2015). As, glucose metabolism is regulated through PI3K/Akt pathway (phosphoinositide-3-kinase protein kinase B pathway) (Wang et al., 2016), WNT signaling has a role in glucose metabolism through PI3K/Akt pathway (Perry et al., 2011; Cisternas et al., 2016). WNT signaling has an important role in the control of energy intake and modulation of the energy balance (Helfer and Tups, 2016).

## PPAR gamma

PPAR gamma is a ligand-activated transcriptional factor that belongs to the nuclear hormone receptor super family. It heterodimerizes with retinoid X receptor (RXR). PPAR gamma is expressed in numerous cell types, such as adipose tissues, muscles, brain, and immune cells. PPAR gamma activates the expression of many genes and regulates glucose homeostasis, insulin sensitivity, lipid metabolism, immune responses, cell fate, and inflammation (Elbrecht et al., 1996; Fajas et al., 1997; Desvergne and Wahli, 1999). PPAR gamma is abundantly expressed in adipose tissue and lower expressed in heart, skeletal muscle, and liver (Canevari et al., 2004; Burkart et al., 2007; Bright et al., 2008). PPAR gamma is low expressed in CNS (central nervous system) and presents in several cell types such as neurons, astrocytes, oligodendrocytes, and microglia (Braissant et al., 1996; Chiang et al., 2010, 2015; Chen et al., 2012). In neurons, PPAR gamma immunoreactivity appears mainly as a nuclear labeling although sometimes cytoplasmic staining is detectable in some cortical neuron (Chiang et al., 2015). PPAR gamma agonists thiazolidinediones (TZDs) improve insulin sensitivity in peripheral tissues (Rangwala and Lazar, 2004) and ameliorate glucose tolerance and insulin sensitivity in type 2 diabetic patients (Picard and Auwerx, 2002). TZDs act on the promoters of glucose transporter (GLUT2) and glucokinase (GK) in pancreatic beta-cells and liver. Abnormalities of PPAR gamma are observed in several pathological states such as cancers, diabetes, obesity, and atherosclerosis. Some TZDs have been used for treating type 2 diabetes. PPAR gamma also plays an important role in regulating cardiovascular rhythms by controlling circadian variations of blood pressure and heart rate through Bmal1 (Wang et al., 2008; Lecarpentier et al., 2010). PPAR gamma agonists could be regulators of glucose metabolism (Janani and Ranjitha Kumari, 2015) given that PPAR gamma is repressed by PI3K/Akt (Berger et al., 2015). Metabolic effects of PPAR gamma agonists are mediated by mitochondrial target of thiazolidinediones, mtot1 and mtot2 which represent the pyruvate transporter (Colca et al., 2013). PPAR gamma agonists have potential glucose-lowering effects (Lavecchia and Di Giovanni, 2015).

## Opposing effects of the canonical WNT/beta-catenin pathway and PPAR gamma

The link between the WNT/beta-catenin pathway and PPAR gamma involves the TCF/LEF beta-catenin domain and a catenin binding domain within PPAR gamma. In numerous mammalian cells, PPAR gamma and WNT/beta-catenin signaling behave in an opposite manner (Gerhold et al., 2002; Girnun et al., 2002; Sharma et al., 2004; Liu et al., 2006; Takada et al., 2009; Lu and Carson, 2010). In some diseases, although the WNT/beta-catenin pathway is downregulated, PPAR gamma appears to be upregulated (Lecarpentier et al., 2014). This has been observed in ARVC (Djouadi et al., 2009), osteoporosis, bipolar disorder, and schizophrenia and certain neurodegenerative diseases (NDs) such as Alzheimer's disease (Vallée and Lecarpentier, 2016). Conversely, in other diseases, WNT/beta-catenin signaling is upregulated while PPAR gamma is downregulated. This is the case in cancers, type 2 diabetes, and certain neurodegenerative diseases (NDs), such as amyotrophic lateral sclerosis (Lecarpentier and Vallée, 2016), Huntington's disease, multiple sclerosis, and Friedreich's ataxia. In several cellular systems, beta-catenin is inhibited by PPAR gamma agonists (Elbrecht et al., 1996; Fajas et al., 1997; Moldes et al., 2003; Zhang et al., 2012). It has also been observed that inhibition of the WNT/beta-catenin pathway induces activation of PPAR gamma (Garcia-Gras et al., 2006).

## Activation of WNT/beta-catenin pathway and inactivation of PPAR gamma in gliomas

WNT/beta-catenin signaling has been activated in cancers (Polakis, 2012a,b). Increased expression of beta-catenin may be due to factors such as mutations in beta-catenin, abnormalities in the beta-catenin destruction complex, mutations in APC, overexpression of WNT ligands, and loss of inhibition or decreased activity of regulatory pathways.

Overexpression of WNT1 and WNT3a in glioma stem cells has been shown in the malignant transformation and progression of high-grade gliomas (Zhang J. et al., 2011; Riganti et al., 2013; Denysenko et al., 2016), WNT2 and WNT5 are also overexpressed in glioma. Beta-catenin is upregulated in glioblastoma tissues compared with normal brain and beta-catenin is associated with glioma progression Yu et al., 2007; Pu et al., 2009; Sareddy et al., 2009; Liu C. et al., 2011; Polakis, 2012b. In malignant astrocytic gliomas, nuclear and nuclear-cytoplasmic positivity of beta-catenin have been shown (Utsuki et al., 2002; Sareddy et al., 2009; Zhang et al., 2009; Yang et al., 2010; Liu X. et al., 2011; Schule et al., 2012). In numerous studies, the nuclear translocation of beta-catenin in glioblastoma has been shown (Sareddy et al., 2009; Yang et al., 2010; Chen et al., 2011; Liu C. et al., 2011; Liu X. et al., 2011; Zhang N. et al., 2011; Kaur et al., 2013; Riganti et al., 2013). The aberrant activation of canonical WNT/beta-catenin pathway contributes to glioma development and malignant progression (Utsuki et al., 2002; Sareddy et al., 2009; Yang et al., 2010; Liu X. et al., 2011; Schule et al., 2012; Yang C. et al., 2012), invasion (Kahlert et al., 2012), and prognostic implications (Liu C. et al., 2011; Rossi et al., 2011; Shi et al., 2012).

In gliomas, PPAR gamma agonists inhibit cell proliferation by induction of cell-cycle arrest in G0/G1 phase (Zang et al., 2003; Liu et al., 2004; Chearwae and Bright, 2008), and reduction of the proportion of cells entering S-phase (Zang et al., 2003; Liu et al., 2004; Chearwae and Bright, 2008). PPAR gamma agonists reduce local tissue invasiveness (Grommes et al., 2006; Papi et al., 2009; Wan et al., 2011), and reduce beta-catenin expression without changing its cellular localization (Wan et al., 2011).

## Aerobic glycolysis in cancer cells: role of the canonical WNT signaling (Thompson, 2014)

Glucose is the major source of energy for mammalian cells, including cancerous cells like gliomas. Glucose is metabolized to generate ATP, through cytosolic glycolysis and oxygen-dependent mitochondrial metabolism, in which most of the reducing potential is the outcome of the TCA cycle. The entry of glucose into the TCA cycle is controlled by PDH. Mitochondrial inactivation in cancer is predominantly due to the inhibition of PDH by PDK (Jha and Suk, 2013).

The role of WNT/beta-catenin signaling in cancer development is now better understood (Bienz and Clevers, 2000). Upregulation of the WNT/beta-catenin pathway via TCF/LEF leads to cell proliferation, EMT, migration, and angiogenesis (Brabletz et al., 2005; Klaus and Birchmeier, 2008; Clevers and Nusse, 2012). In cancer cells, overactivation of the WNT/beta-catenin pathway induces aerobic glycolysis. This allows glucose utilization for cell proliferation (Pate et al., 2014). Thus, in a large part, glucose supply is fermented to lactate regardless of oxygen availability. This phenomenon is referred to as aerobic glycolysis or the Warburg effect (Warburg, 1956).

In cancer, the behavior of two key enzymes in glucose metabolism is modified leading to the Warburg effect. Activation of PDK-1 is required for the Warburg aerobic glycolysis. Upregulation of WNT/beta-catenin signaling activates both PDK-1 and MCT-1. PDK1, a major regulator of glucose metabolism, phosphorylates the PDH, which is inhibited and largely prevents the conversion of pyruvate into acetyl-CoA in mitochondria (Roche et al., 2001). In colon cancer, PDK-1 is upregulated (Koukourakis et al., 2006; Pate et al., 2014), so that the conversion of pyruvate into acteyl-CoA in mitochondria is proportionally diminished with a consequent reduction of acetyl-CoA entering the tricarboxylic acid (TCA) cycle. This induces aerobic glycolysis despite the availability of oxygen. PDK-1 has also been observed to be upregulated in several other cancers (Wigfield et al., 2006; Baumunk et al., 2013). Cytosolic pyruvate is converted into lactate through activation of LDH-A. Upregluation of both lactic dehydrogenase-A (LDH-A) and lactate transporter (MCT-1) results in pyruvate being diverted toward the formation of lactate and the secretion of the latter outside of the cell, which favors angiogenesis (Hunt et al., 2007) and ultimately leads to anabolic production of biomass, nucleotide synthesis (De Berardinis et al., 2008; Vander Heiden et al., 2009). The Warburg effect partly shunts the TCA cycle leading to aerobic glycolysis, which is less efficient in terms of ATP production. The most cost effective way production ATP is via glucose oxidation (ATP/O_2_ = 6.4), since the pathway via free fatty acid beta-oxidation is less efficient (ATP/O_2_ = 5.6). This takes about 11% more O2 to produce the same amount of ATP from fatty acids as it does from glucose. Moreover, PDK-1 and 2 enhance angiogenesis (McFate et al., 2008; Sutendra et al., 2013). Blocking WNT reduces the PDK-1 level via the transcription regulation and reduces *in vivo* tumor growth. Conversely, PPAR gamma activation selectively decreases PDK mRNA (Abbot et al., 2005). PDKs allow metabolic flexibility (Zhang et al., 2014) and are transcriptionally regulated by insulin, glucocorticoids, thyroid hormone and fatty acids (Lee, 2014). Several diseases presenting PDK abnormalities are often associated with type 2 diabetes, obesity, metabolic disorders, cardiomyoptahies, neuropathies, and cancers.

In colon cancer, activation of WNT/beta-catenin signaling proportionally decreases the oxidative metabolism in the TCA cycle and promotes cell proliferation (Pate et al., 2014). In addition, the WNT/beta-catenin pathway induces the transcription of genes involved in cell proliferation, particularly cyclin D1 and c-Myc operating through the G1 phase (Osthus et al., 2000; Nusse, 2005; Niehrs and Acebron, 2012). c-Myc activates aerobic glycolysis and glutaminolysis and favors nucleotide synthesis (Wise et al., 2008). Moreover, c-Myc increases the hypoxia-inducible factor 1 alpha (HIF-1 alpha) with controls PDK-1 (Kim et al., 2007). Part of the pyruvate entering the TCA cycle is converted into citrate, which promotes protein and lipid synthesis. Cellular accumulation of metabolic intermediates (aspartate, serine, glycine, and ribose) allows *de novo* nucleotide synthesis, which contributes to growth and proliferation. Angiogenesis is also favored by production of lactate (Lu et al., 2002).

Phosphofructokinase (PFK), an allosteric enzyme, is responsible for glycolytic oscillations. PFK can lead to instabilities beyond which a new state can be organized in time and in space (Goldbeter, 1973). A positive feedback is responsible for periodic behavior. These far-from-equilibrium oscillatory mechanisms come within the field of dissipative structures initially described by Prigogine (Prigogine and Nicolis, 1971). Elevated PFK-1 activity is characteristic of cancer cells and is induced in response to ontogenesis (Mor et al., 2011).

## Canonical WNT pathway and glucose

Cancer cells are characterized by increased glucose consumption. High serum glucose levels may modulate cancer-related processes. Glucose itself can directly impact the canonical WNT pathway (Chocarro-Calvo et al., 2013). High glucose level enhances the nuclear translocation of beta-catenin in response to WNT activation. In cancer cells, glucose-induced beta-catenin acetylating favors the WNT pathway.

## Aerobic glycolysis in gliomas (cf. Figure 1)

Glucose metabolism has been identified as important biological markers in glioma cells for the progression of gliomas (Morfouace et al., 2012). Glycolytic metabolism is upregulated in gliomas (Mineura et al., 1986; Oudard et al., 1996). Activation of PDK in gliomas leads to shunt pyruvate from the mitochondria (Jha and Suk, 2013). Glioma cells suffer from nutrient deprivation and are more susceptible to cytotoxic killing than normal astrocytes (Spagnolo et al., 2007). This effect is mediated by reactive oxygen species produced by mitochondria (Ahmad et al., 2005). Numerous studies on gliomas have shown the dependence of glioma cells on glycolysis as primary source of energy (Maurer et al., 2011). Upregulation of glycolysis shows increasing glucose consumption and is defined as a feature of primary and metastatic cancers (Gatenby and Gillies, 2004). High-grade gliomas have high rates of glycolysis and lactate production (Jha and Suk, 2013). Overexpression of MCTs, especially MCT-1, has been reported in neoplasic human cells, including the most aggressive forms of glioma cells tumors (Galeffi and Turner, 2012). MCT-1 immunoreactivity is significantly higher in high-grade glioma than low-grade (Froberg et al., 2001). The overexpression of MCTs is likely an adaptive response of tumor expansion at different levels. It helps glioma cells to maintain a high rate of glycolysis by exporting lactate to extracellular space. Glioma cells have an increase of lactate concentration in intracellular, which are accompanied by a progressive inhibition of the TCA cycle (Bouzier-Sore et al., 2001).

**Figure 1:**
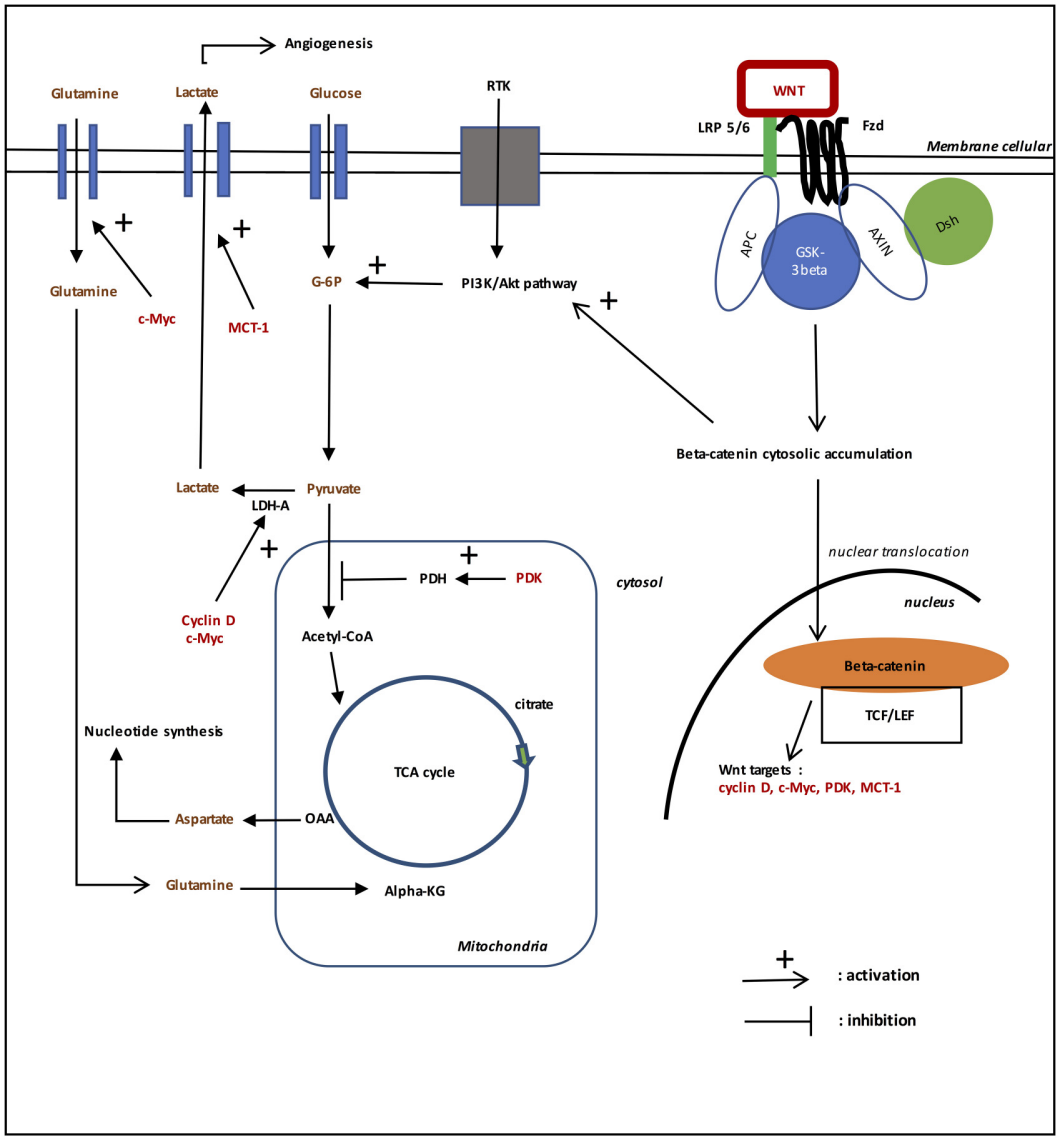
Role of WNT/beta-catenin pathway on aerobic glycolysis in gliomas. In the presence of WNT ligands (“on state”), WNT binds both Frizzled and LRP 5/6 receptors to initiate LRP phosphorylation of the Axin/APC/GSK-3beta complex. Beta-catenin phosphorylation is inhibited which prevents its degradation in the proteasome. Beta-catenin accumulates in the cytosol and then translocates to the nucleus to bind TCF-LEF co transcription factors. This induces the WNT-response gene transcription (PDK, c-Myc, cyclin D, MCT-1). Glucose itself activates the WNT pathway. MCT-1 favors lactate extrusion out of the cytosol which favors angiogenesis. WNT/beta-catenin pathway stimulates tyrosine kinase receptors (TKRs) activation. Activation of PI3K/Akt increases glucose metabolism. Akt-transformed cells protect against reactive oxygen species stress (ROS stress) by inducing HIF-1alpha, which suppresses glucose entry into the TCA cycle. HIF-1alpha induced PDK1 phosphorylates PDH, which resulting in cytosolic pyruvate being shunted into lactate through induction of LDH-A. PDK inhibits the PDH complex in mitochondria, thus pyruvate cannot be fully converted into acetyl-CoA and enter the TCA cycle. c-Myc and cyclin D also activates LDH-A which converts cytosolic pyruvate into lactate. c-Myc increases glutamine entry in the cytosol and mitochondria. c-Myc-induced glutamine enhances aspartate and nucleotide synthesis.

## Role of PI3K-Akt pathway in aerobic glycolysis in gliomas (cf. Figure 1)

EGFR (Epidermal growth factor receptor) is a type of receptor tyrosine kinase (RTK) playing a central role in cell division, migration, adhesion, differentiation, and apoptosis (Chakravarti et al., 2004; Mellinghoff et al., 2005). EGFR overexpression, occurring in 30–70% of primary glioblastomas is the most frequent mutation (Popescu et al., 2016). EGFR activation by binding PI3K (phosphoinositide-3-kinase), Akt (protein kinase B), STAT (signal transducer and activator of transcription) etc. modulates cell proliferation, differentiation and survival (Park et al., 2004; Seshacharyulu et al., 2012). WNT/beta-catenin pathway stimulates tyrosine kinase receptors (TKRs) activation in gliomas (Gruetter, 2003; Yang et al., 2016). Downregulation of beta-catenin reduces the expression of EGFR, Akt1, Akt2, and phosphorylates Akt (Park et al., 2004; Yue et al., 2010; Zhang N. et al., 2011).

EGFR activates PI3K. PIP2 (phosphatidylinositol-3,4-biphosphate) is converted in PIP3 (phosphosphatidyk-3,4,5-triphosphates) by PI3K and back by phosphatase and tensin homolog (PTEN) (Yue et al., 2010; Sami and Karsy, 2013). PI3K activates Akt signaling while PTEN suppresses it, Akt is activated by phosphorylation. Increasing Akt signaling inactivates GSK-3beta by phosphorylating the protein at Ser-9, this leads to nuclear translocation and stabilization of beta-catenin (Paw et al., 2015). PI3K/Akt signaling pathway is involved in cell proliferation, cell survival, and endothelial cell migration (Sami and Karsy, 2013; Xu et al., 2013; Wang et al., 2014). PI3K/Akt pathway regulates beta-catenin stability, localization, transcriptional activity (Paw et al., 2015), and the expression of its downstream genes (such as cyclin D1, c-Myc) (Ji et al., 2011).

Activation of PI3K/Akt increases glucose metabolism. Hyper activation of PI3K/Akt pathway is associated with an increased rate of glucose metabolism in tumor cells (Reuter et al., 2010). Akt signaling directly acts on glycolysis in cancer cells. Akt regulates the localization of GLUT1 in the plasma membrane and hexokinase expression. It also activates phosphofructokinase-1 (PFK-1), which directly phosphorylates PFK2, leading to the production of fructose-2,6-biphosphate, an activator of PFK1. Akt activation causes an increase in aerobic glycolysis effect in cancer. PI3K/Akt pathway promotes cell survival, cell growth, cell proliferation, cell migration and angiogenesis in response to extracellular signals including hormones and growth factors. Through phosphorylation of GSK-3beta, PI3K/Akt favors the G1-S phase of the cell cycle. GSK-3beta phosphorylation decreases the degradation of beta-catenin in the proteasome. Thus, TCF/LEF transcription factor is activated, which in turn favors transcription of the target gene cyclin D1 (Alao, 2007). PI3K/Akt contributes to angiogenesis by acting on the vascular endothelial growth factor in endothelial cells and on the endothelial nitric oxide synthase; this activates vasodilatation and vascular remodeling (Manning and Cantley, 2007).

Akt-transformed cells protect against reactive oxygen species stress (ROS stress) by inducing HIF-1alpha, which suppresses glucose entry into the TCA cycle (Lum et al., 2007). HIF-1alpha induced PDK1 (pyruvate dehydrogenase kinase 1) phosphorylates PDH (pyruvate dehydrogenase), which resulting in cytosolic pyruvate being shunted into lactate through induction of LDH-A (lactate dehydrogenase A) (Suda et al., 2011). Activation of PI3K/Akt results in aerobic glycolysis. Blockage of EGFR/PI3K/Akt signaling axe could be an interesting therapeutic perspective to improve the survival of patients with glioblastoma (Tanase et al., 2013).

## Prostaglandins, WNT/beta-catenin pathway, and PPAR gamma in gliomas

Several studies have established the role of prostaglandin E2 (PGE2) by activating the WNT/beta-catenin pathway. The link between PGE2 and the canonical WNT pathway suggests that chronic inflammation induced by a prolonged increase of PGE2 could lead to activation of WNT signaling resulting in cell proliferation and cancer. PGE2 enhances the beta-catenin-dependent transcription (Castellone et al., 2005; Suda et al., 2011). PGE2 could promote cancer cells growth through the beta-catenin pathway. Thus, blockage of WNT/beta-catenin signaling can be of interest for gliomas treatment.

NSAIDs (non-steroidal anti-inflammatory drugs) can reduce nuclear beta-catenin levels and induce beta-catenin degradation (Rice et al., 2003; Tinsley et al., 2011; Gurpinar et al., 2014). Sulindac, exisulind, and celecoxib (NSAIDs) decrease beta-catenin level and inhibit transcriptional activity of the beta-catenin/TCF/LEF complex (Thompson et al., 2000; Maier et al., 2005). NSAIDs inhibit glioma invasion *in vitro* by dephosphorylation of Akt, which causes a decrease in MMP-2 gene expression and activity (Lee et al., 2005; Paw et al., 2015). NSAIDs can eliminate stem cells with nuclear beta-catenin and aberrant WNT signaling in APC Min mice and in human colonic polyps through the induction of apoptosis (Jiang et al., 2012). Ibuprofen (which belong to the group of NSAIDs) have significant effects on glioma cell proliferation and apoptosis (Ribeiro et al., 2008; Benadiba et al., 2010), PGE2 alters proliferative, apoptotic and migratory, and migratory properties of human glioma cells (Gomes and Colquhoun, 2012).

PGE2 modulates WNT activity hematopoietic stem cell (HSC) in zebrafish. Inhibition of PGE2 synthesis blocks alterations in HSC induced by WNT. PGE2 modifies the WNT signaling cascade at the level of beta-catenin degradation through the cAMP/PKA pathway. WNT activation in stem cells requires PGE2 (Goessling et al., 2009). Dimethyl-prostaglandin E2 increases HSC *in vivo*. In addition, dimethyl-prostaglandin E2 leads to the formation of components of the WNT pathway (Li et al., 2014). WNT signaling upregulates interleukin (IL)-7R and IL-2Rbeta. In neuroectodermal (NEC-4C) stem cells, PGE2 interacts with canonical WNT signaling through PKA and PI3K (Wong et al., 2014). In WNT-induced cells, beta-catenin is increased and the WNT-targets gens (Ctnnb1, Ptgs2, Ccnd1, Mmp9) are significantly upregulated after PGE2 used. COX-2/PGE2 have a functional role in glioma (Chiu et al., 2010). Specific COX-2 inhibitor inhibited the proliferation and invasion of cultured glioma cell lines. PPAR gamma and pro-inflammatory enzyme pathways are interrelated. Decreased expression of PPAR gamma and high levels of cyclooxygenase-2 (COX-2) have been reported in many cancers (Hazra et al., 2008). TZDs decrease COX-2, inhibit growth of non-small-cell lung cancer cells *in vitro*, and block tumor development. TZDs diminish COX-2and PGE2 through PPAR gamma. The PPAR gamma activator 15dPGJ2 pays an anti-inflammatory role in PPAR gamma-dependent manner, decreasing COX-2, PGE2, and iNOs expression (Mendez and LaPointe, 2003).

## Circadian rhythms (CRs), gliomas, metabolism, and thermodynamics (cf. Figure 2) (Savvidis and Koutsilieris, 2012)

The circadian “clock,” located in the hypothalamic suprachiasmatic nucleus (SCN), is known to drive numerous biologic processes in the body. CRs can be defined as endogenous, entrainable free-running periods that last ~24 h. CRS are far-from-equilibrium dissipative structures and are due to a negative feedback produced by a protein on the expression of its own gene (Goodwin, 1965; Hardin et al., 1990). They operate in far-from-equilibrium manner if affinity of the studied system is RT (R is the universal gas constant and T is the absolute temperature), and generate order spontaneously by exchanging energy with their external environment (Prigogine et al., 1974; Goldbeter, 2002). In mammals, CRs involve several major critical transcription factors Clock (Circadian locomotors output cycles kaput), Bmal1 (brain and muscle aryl-hydrocarbon receptor nuclear translocator-like 1), Per1 (Period 1), Per2 (Period 2), Per3 (Period 3), Cryptochrome (Cry 1 and Cry 2) (Gekakis et al., 1998; Hogenesch et al., 1998). Transcription/translation auto regulatory feedback loops with both activating and inhibiting pathways are involved in CRs (Reppert and Weaver, 2002; Schibler and Sassone-Corsi, 2002). Clock and Bmal1 heterodimerize and initiate transcription of target genes, such as Period (Per1 and Per2) and Cryptochrome (Cry1 and Cry2) (Ko and Takahashi, 2006). A negative feedback is achieved by Per/Cry heterodimers that translocate back to the nucleus to repress their own transcription by acting on the Clock/Bmal1 complex (Ko and Takahashi, 2006). Clock/Bmal1 heterodimers activate transcription of retinoic acid-related orphan nuclear receptors, such as Rev-Erbs and RORs (retinoic acid receptor-related orphan receptors). In feedback, RORs activate transcription of Bmal1, whereas Rev-Erbs repress the transcription process (Ko and Takahashi, 2006). RORs are regulation factors downstream of the WNT/beta-catenin pathway (Chen, 2004). The circadian oscillation of Bmal1 is both positively and negatively regulated by RORs and Rev-Erbs.

**Figure 2:**
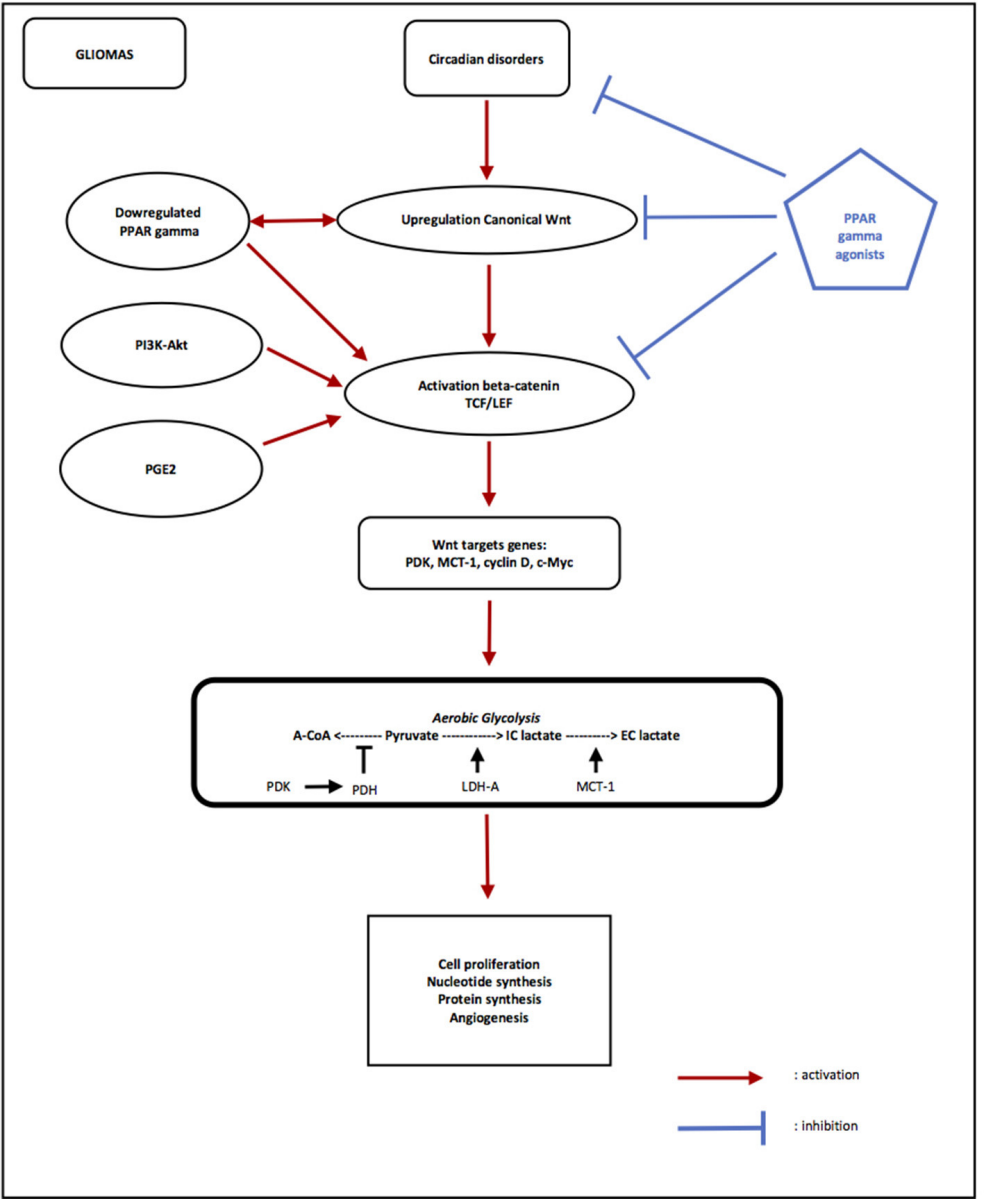
Opposing effects of PPAR gamma and canonical WNT/beta-catenin signaling in gliomas. Circadian rhythms disorders are observed in Gliomas, with decreasing of Per and increasing of Bmal1 and Clock. Overexpression of Bmal1 induces activation of WNT pathway. Upregulation of canonical WNT and downregulation of PPAR gamma are observed in gliomas. PI3K/Akt pathway and PGE2 also activate beta-catenin. The activation of beta-catenin induces transcription of WNT targets genes such as PDK, MCT-1, c-Myc, and cyclin D. PDK inhibits the PDH complex in mitochondria, thus pyruvate cannot be fully converted into acetyl-CoA and enter the TCA cycle. c-Myc and cyclin D activates LDH-A which converts cytosolic pyruvate into lactate. MCT-1 favors lactate extrusion out of the cytosol which favors angiogenesis. This effect is called aerobic glycolysis. Activation of WNT/beta-catenin pathway induces aerobic glycolysis, and then results in cell proliferation, nucleotide synthesis, protein synthesis and angiogenesis in gliomas. PPAR gamma agonists induce activation of Bmal1 and the formation of heterodimers Clock/Bmal1, and then inhibit the WNT pathway. PPAR gamma agonists directly inhibit activation of beta-catenin and its nuclear translocation and then inhibit transcription of WNT targets genes. Using PPAR gamma agonists may interest to stop gliomas progression through inhibition of aerobic glycolysis via inactivation of canonical WNT/beta-catenin pathway.

CRs govern numerous physiological and metabolic functions (Sahar and Sassone-Corsi, 2009). Thus, CRs are observed in sleep-awake and feeding patterns, energy metabolism, body temperature, hormone secretion, heart rate, and blood pressure. Following epidemiological and genetic probes, it has been suggested that disruption of CRs may be directly linked to cancer, leading to aberrant cellular proliferation (Wood et al., 2009). Since numerous connections between the circadian clock and cellular metabolism have been reported, it is through that the abnormal metabolism observed in cancer may be a consequence of disrupted CRs. Altered links between the circadian clock and cellular metabolism have been discovered and might be regulated by chromatin remodeling. CRs within the cell regulate the timing of many important life cycles (Cao et al., 2015). The phase diffusion constant depends on the free-energy dissipation per period. Oscillations are driven by multiple irreversible cycles that hydrolyze fuel molecules such as ATP. The free energy consumed per period is proportional to the number of phase coherent periods.

Circadian genes may control cell cycle progression via WNT pathway which contains putative Bmal1/clock-binding sites within its promoters (WNT10a, beta-catenin, Dsh2, TCF3) (Soták et al., 2014). A Bmal1 knockdown inhibits WNT signaling expression and activity (Guo et al., 2012). Lower levels of WNT-related genes are associated with Bmal1 knockdown compared to the expression of WNT-related genes factors varies with a 12-h period in wild-type mice (Janich et al., 2011). Bmal1 knockout mice show lower levels of WNT-related genes (Yasuniwa et al., 2010). Circadian disruption accelerates tumor growth through the WNT pathway in nude mouse xenograft models (Reppert and Weaver, 2002). Cell proliferation and cell cycle progression may regulate by circadian clock gene Bmal1 through activation of canonical WNT/beta-catenin pathway (Lin et al., 2013). Bmal1 may reduce beta-catenin degradation and may enhance its transcription. Bmal1 represses WNT inhibitors or degradation factors of beta-catenin, like GSK-3beta (Coyle, 2007; Sahar et al., 2010).

Beta-catenin induces Per2 degradation altering circadian clock gene in intestinal mucosa of ApcMin/+ mice (Yang et al., 2009). A decreased expression of Per1 and or Per2 has been reported in numerous cancers: breast cancer (Winter et al., 2007), prostate cancer (Cao et al., 2009), pancreatic cancer (Suzuki et al., 2008), colorectal cancer (Mostafaie et al., 2009), chronic myeloid leukemia (Yang et al., 2011), gliomas (Fujioka et al., 2006; Xia et al., 2010), and intestinal epithelial neoplastic transformation (Yang et al., 2009). Overexpression of Per1 and Per2 inhibits the growth of cancers cells (Gery et al., 2006; Hua et al., 2006) and increases apoptosis in tumor cells (Fu et al., 2002; Gery et al., 2006; Hua et al., 2006; Sun et al., 2010). Per1 and Per2 maintain the circadian rhythm of cells and sustain the normal cell cycle by regulating the expression of cell-related genes such as p53 and c-Myc (Duffield et al., 2002; Fu et al., 2002; Sancar et al., 2004). In normal circumstances, the core circadian genes work in accurate feedback loops and keep the molecular clockworks in the SCN. They allow controlling peripheral clocks (Reppert and Weaver, 2002; Schibler and Sassone-Corsi, 2002). The levels of mRNAs and proteins of circadian genes oscillate throughout the 24 h period, exceptions of Clock (Reppert and Weaver, 2001). Prevents studies have demonstrated the expression of mClock as a nuclear antigen in the SCN (Maywood et al., 2003). Circadian clocks gene involves in gliomagenesis (Li et al., 2013). The expression of Clock gene in the high-grade gliomas was found to be significantly higher than the low-grade gliomas and non-gliomas (Chen et al., 2013). Clock gene is increased in grade III and IV glioma tissues cell lines (Li et al., 2013). NF-kappaB activity is reduced and NF-KappaB target genes are repressed after Clock knockdown. An aberrant expression of Clock may disrupt the NF-kappaB pathway in glioma (Li et al., 2013). Abnormalities in Per1 and Per2 expression are associated with the occurrence of gliomas (Xia et al., 2010). Deregulation expression of c-Myc is suggested as a key factor leading to tumor development in Per2 mutant mice (Fu et al., 2002). Overexpression of Per2 in irradiated glioma induces a decreased of c-Myc mRNA and protein levels (Gery et al., 2006). The overexpression of Per2 induces Bmal1 expression and then increases intracellular levels of Bmal1/Clock proteins, in addition to repressing c-Myc (Fu et al., 2002). The p53 protein binds the c-Myc promoter and represses it (Ho et al., 2005). Overexpression of Per2 promotes apoptosis in glioma tissue by downregulating c-Myc and upregulating p53 (Zhanfeng et al., 2015). Melatonin, which regulates circadian rhythms, has been demonstrated to significantly reduce damage-induced apoptosis in astrocytoma cells (Radogna et al., 2009). Circadian genes may have a potential impact on glioma survival; genetic variation in the circadian pathway is associated with risk or outcome of glioma (Madden et al., 2014).

PPAR interferes with the mammalian clock and energy metabolism (Chen and Yang, 2014). PPARs are rhythmically expressed in mammalian tissues (Yang et al., 2006), and directly interact with the core clock genes. PPAR gamma exhibits variations in diurnal expression in mouse fat, liver, and blood vessels (Yang et al., 2006; Wang et al., 2008). Deletion of PPAR gamma in mouse impairs diurnal rhythms (Yang G. et al., 2012). PPAR gamma plays an important role in the coordinated control of circadian clocks, metabolism, and cardiac performance (Yang G. et al., 2012). PGC-1 alpha, a transcriptional co-cativator that regulates energy metabolism, is rhythmically expressed in liver and skeletal muscle of mice. PGC-1 alpha upregulates the expression of clock genes Bmal1 and Rev-Erb alpha. Mice lacking PGC-1alpha show changes in CRs and metabolism (Liu et al., 2007). PGC-1 alpha acts as a stress sensor in cancer cells. In maintaining metabolic homeostasis, PGC-1 alpha favors cancer cell survival and tumor metastasis (Tan et al., 2016). PPAR gamma agonists activate Bmal1 and the formation of heterodimers Clock/Bmal1 (Wang et al., 2008, 2010). Curcumin activates Bmal1 through stimulation of PPAR gamma and could be a promising phytochemical treatment for gliomas (Sarma et al., 2016).

## Conclusion

Gliomas exhibit thermodynamic and metabolic alterations and abnormal circadian rhythms with an increasing entropy rate. In gliomas, the canonical WNT/beta-catenin pathway is upregulated, while PPAR gamma is downregulated. The two systems act in an opposite manner. Overactivation of the WNT pathway results in cell proliferation, due to the activation of target genes of beta-catenin, such as cyclin D1 and c-Myc. This activation of WNT pathway also promotes protein synthesis and angiogenesis. PDK and MCT-1 are also target genes of beta-catenin, explaining the decrease in the transformation of pyruvate into acetyl-CoA in mitochondria and the formation of intracellular lactate, which will be extruded out the cell. This is referred to as aerobic glycolysis or the Warburg effect. Circadian rhythms, dissipative structures, which are governed by the laws for far-from-equilibrium thermodynamics are disrupted in gliomas. They are influenced by both the WNT/beta-catenin pathway and PPAR gamma. Changes in thermodynamics, metabolism, and circadian rhythms are tightly linked in gliomas.

## Author contributions

All authors listed, have made substantial, direct and intellectual contribution to the work, and approved it for publication.

### Conflict of interest statement

The authors declare that the research was conducted in the absence of any commercial or financial relationship that could be construed as a potential conflict of interest. The reviewer DJRL and handling Editor declared their shared affiliation, and the handling Editor states that the process nevertheless met the standards of a fair and objective review.

## Abbreviations

Acetytl-coA: Acetyl-coenzyme A
APC: Adenomatous polyposis coli
ARVC: Arrthymogenic right ventricular dysplasia/cardiomyopathy (ARVC)
Bmal1: Brain and muscle aryl-hydrocarbon receptor nuclear translocator-like 1
Clock: Circadian locomotor output cycles kaput
COX-2: Cyclooxygenase-2
Cry: Cryptochrome
Dsh: Disheveled
EMT: Epithelial-mesenchymal transition
Fzd: Frizzled
GK: Glucokinase
GLUT: Glucose transporter
GSK-3beta: Glycogen synthase kinase-3beta
HSC: Hematopoietic stem cell
LDH: Lactate dehydrogenase
LRP 5/6: Low-density lipoprotein receptor-related protein 5/6
MCT-1: Monocarboxylate lactate transporter-
NSAID: Nonsteroidal anti-inflammatory drug
NDs: Neurodegenerative diseases
Per: Period
PPAR gamma: Peroxisome proliferator-activated receptor gamma
PGC-1alpha: Peroxisome proliferator-activated receptor-gamma coactivator-1 alpha
PI3K-Akt: Phosphatidylinositol 3-kinase-protein kinase B
PFK-1: Phosphofructokinase-1
PGE2: Prostaglandin E2
PDH: Pyruvate dehydrogenase complex
PDK: Pyruvate dehydrogenase kinase
RTK: Receptor tyrosine kinase
TCF/LEF: T-cell factor/lymphoid enhancer factor
TZD: Thiazolidinedione
TCA: Tricarboxylic acid.
